# Supplementary material for: Contribution of GATA6 to homeostasis of the human upper pilosebaceous unit and acne pathogenesis
Source: Nat Commun. 2020 Oct 20;11:5067. doi: 10.1038/s41467-020-18784-z (PMC7575575; doi:10.1038/s41467-020-18784-z)
Supplement: Supplementary file 1 — Supplementary Information [file 41467_2020_18784_MOESM1_ESM.pdf]

Contribution of GATA6 to homeostasis of the human upper pilosebaceous unit and acne pathogenesis

Oulès et al

Supplementary Information PDF

Supplementary Fig. 1

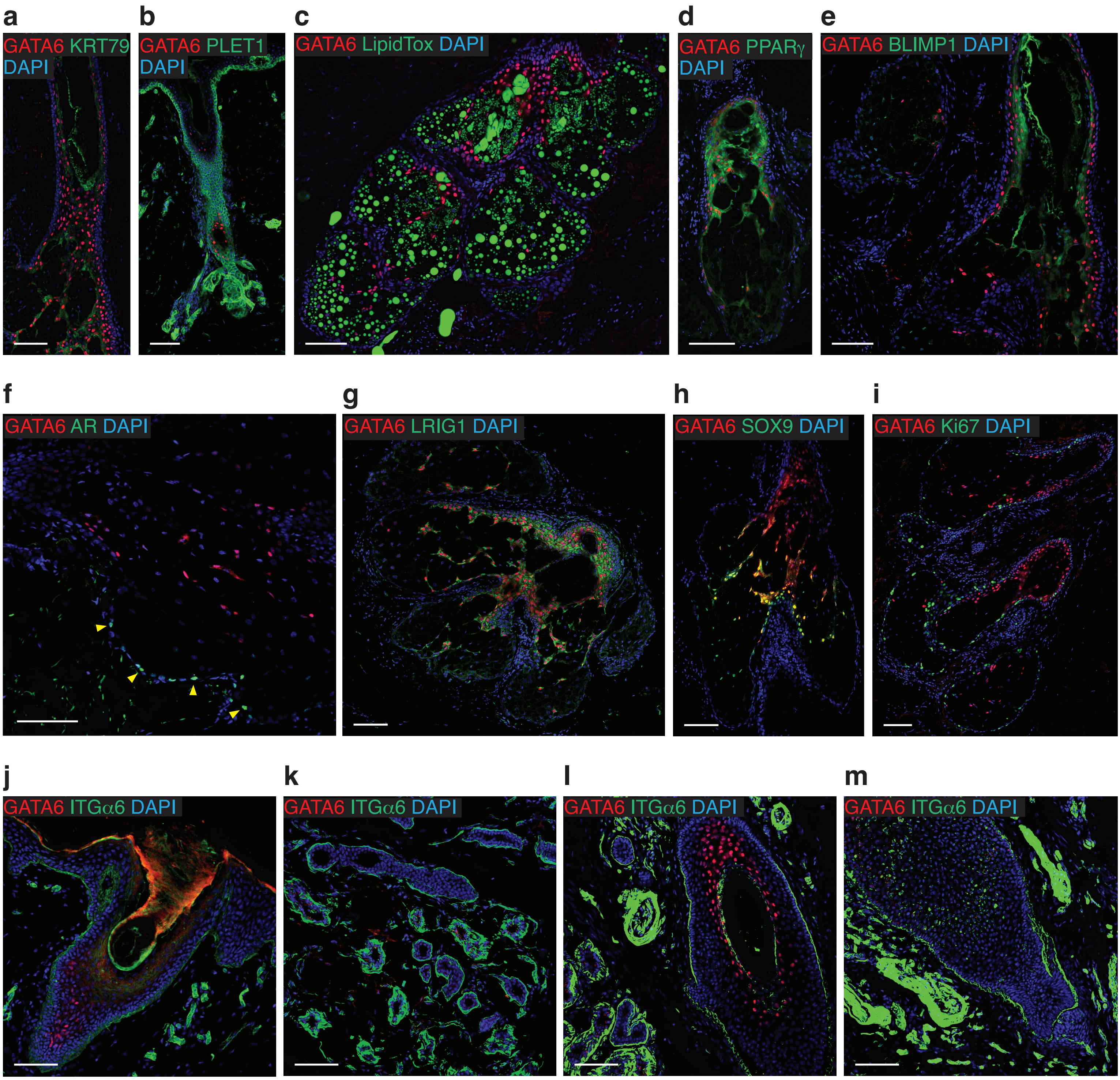

Supplementary Fig. 1: Immuno-profiling of the human upper pilosebaceous unit.
(**a-i**) Back skin sections from a 60 year old male were stained for GATA6 (a-i) and KRT79 (a), PLET1 (b), LipidTOX (c), PPARγ (d), BLIMP1 (e), AR (f), LRIG1 (g), SOX9 (h), and Ki67 (i). Yellow arrowheads show AR+ GATA6- sebocytes in (f). (**j-m**) Abdominal skin sections from a 47 year old male patient were labelled with antibodies against GATA6 and ITGα6. Specific epidermal compartments are shown: IFE and upper INF (j), sweat glands (k), sweat glands and lower INF (l), and lower HF (m). (**a-m**) Nuclei were counterstained with DAPI. Data are representative of two independent experiments. Scale bar: 100 µm.

Supplementary Fig. 2

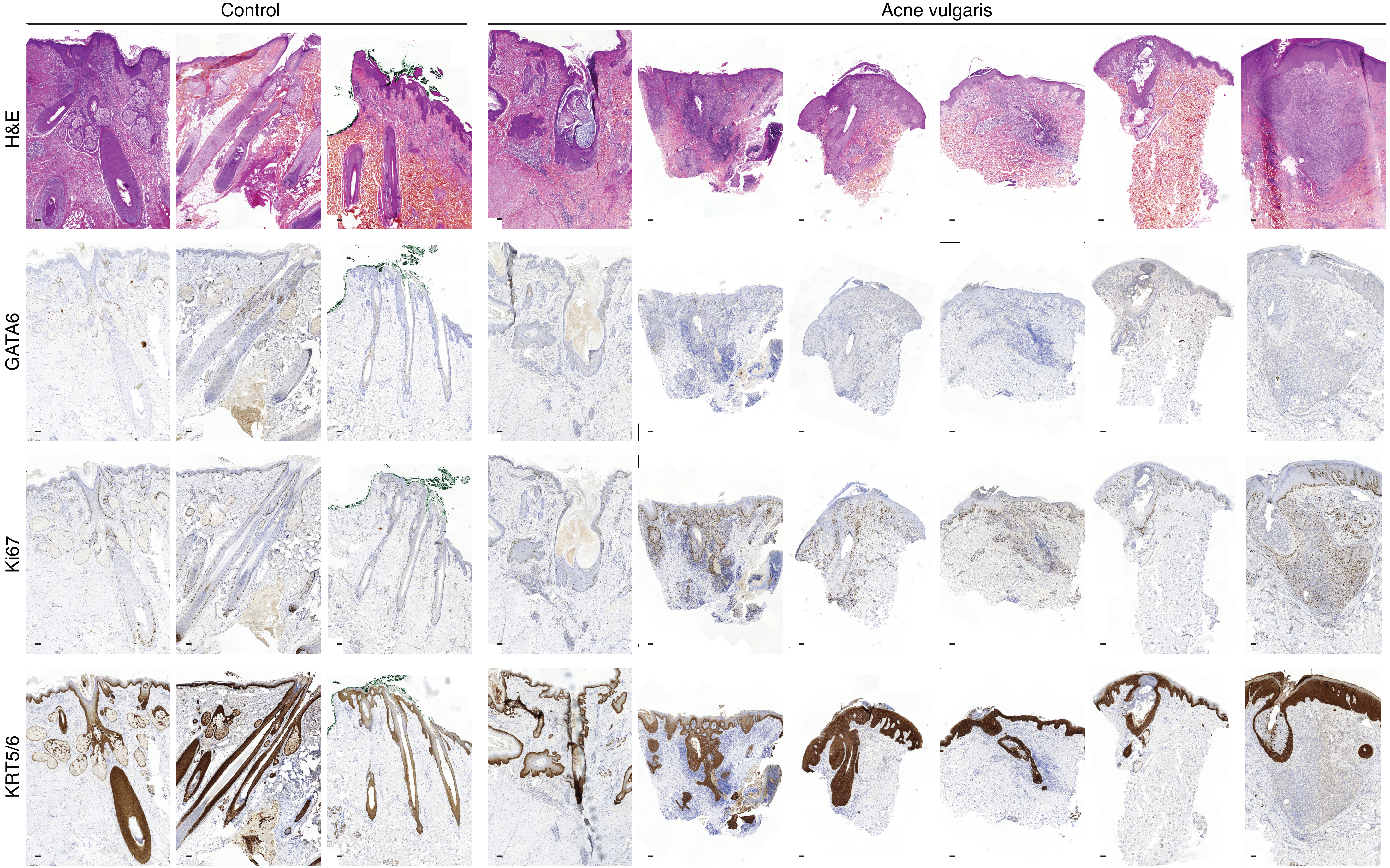

Supplementary Fig. 2: GATA6 expression is reduced in acne patients compared to healthy skin.
3 healthy skin and 6 acne skin sections were stained with Haematoxylin and eosin (H&E), or labelled with antibodies against GATA6, Ki67 and KRT5/6 (brown labelling). Scale bar: 100 μm.

Supplementary Fig. 3

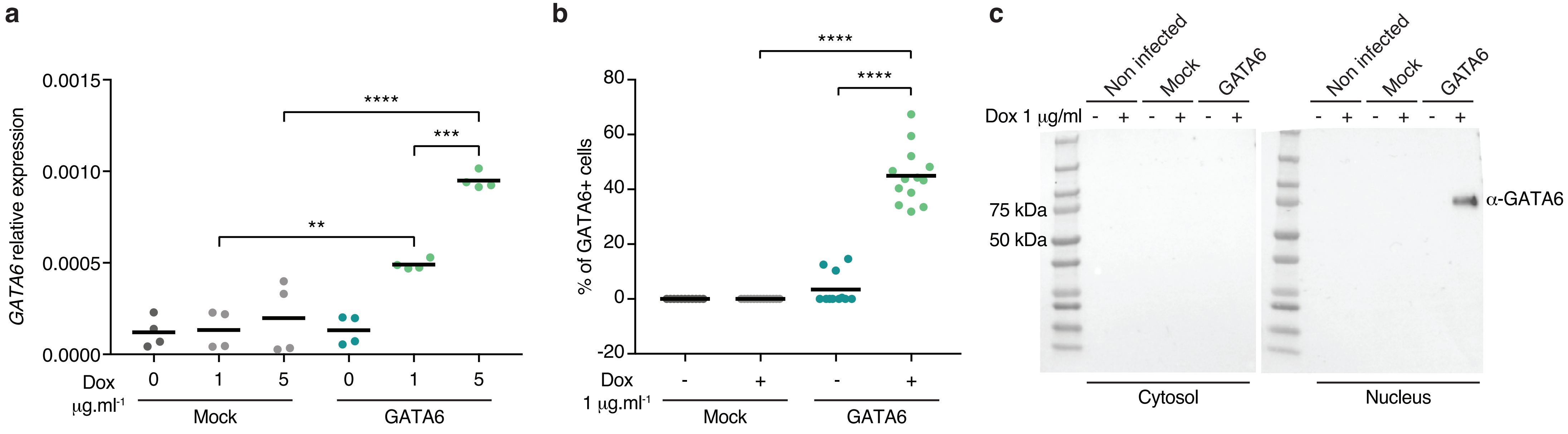

Supplementary Fig. 3: Lentivirus-mediated GATA6 overexpression in human keratinocytes.
(**a**) Dose-response effect of Dox treatment on GATA6 expression. Mock or GATA6-infected keratinocytes were treated with Dox at the indicated concentrations for 48 h. GATA6 mRNA was measured by RT-qPCR (n=4/condition). (**b**) Keratinocytes were treated with 1 $\mu g.ml^{-1}$ Dox for 48 h and nuclear GATA6+ cells were quantified (n=11 for Vehicle conditions, n=13 for Dox condition). (**c**) Cytosolic and nuclear fractions were prepared from non-infected, Mock- or GATA6- infected keratinocytes treated or not with 1 µg/ml Dox for 16 h. Western blotting was performed with a GATA6 antibody. Non-cropped membranes are shown with molecular weight markers. Data are representative of one independent experiment. (**a-b**) Data are mean and individual values from two (a) or three (b) independent experiments corresponding to n replicates. Statistical analysis was performed with ordinary one-way ANOVA. (**) p-value < 0.005; (***) p-value < 0.0005; (****) p-value < 0.00005. Source data are provided as a Source Data file.

Supplementary Fig. 4

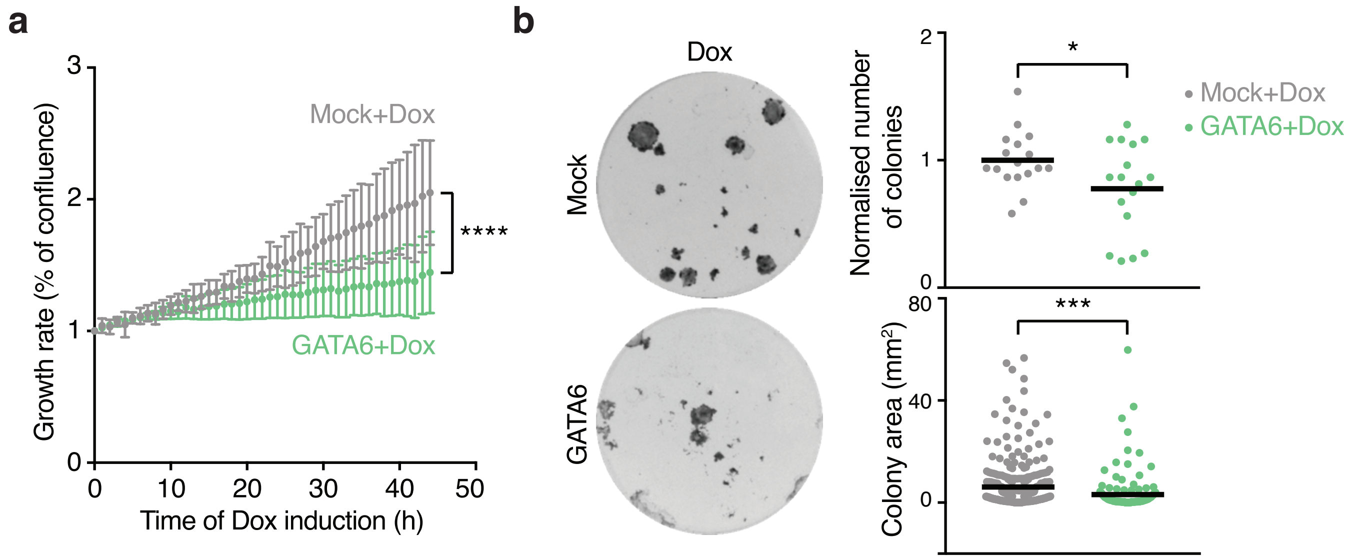

Supplementary Fig. 4: GATA6 overexpression decreases proliferation and colony formation in IFE keratinocytes.
(**a**) Proliferation of IFE keratinocytes assessed as % confluence using an Incucyte video-microscope (one image/h). Values at each time point were normalised to the first scan point of the Mock+Dox condition (n=3/condition; p<0.000001). Cells were treated with 1 µg.ml^{-1} Dox. (**b**) Number of colonies (normalised to the number of colonies in Mock+Dox condition) and colony area of IFE keratinocytes overexpressing GATA6 (n=17/condition; p=0.039614 and p=0.000240 respectively). Representative dishes are also shown. (**a-b**) Data are presented as mean ± SD (a) or mean with individual values (b) and were obtained from three (a) or four (b) independent experiments corresponding to n replicates. Statistical analyses were performed with linear regression (a) or two-tailed unpaired t-test (b). (*) p-value < 0.05; (***) p-value < 0.0005; (****) p-value < 0.00005. Source data are provided as a Source Data file.

Supplementary Fig. 5

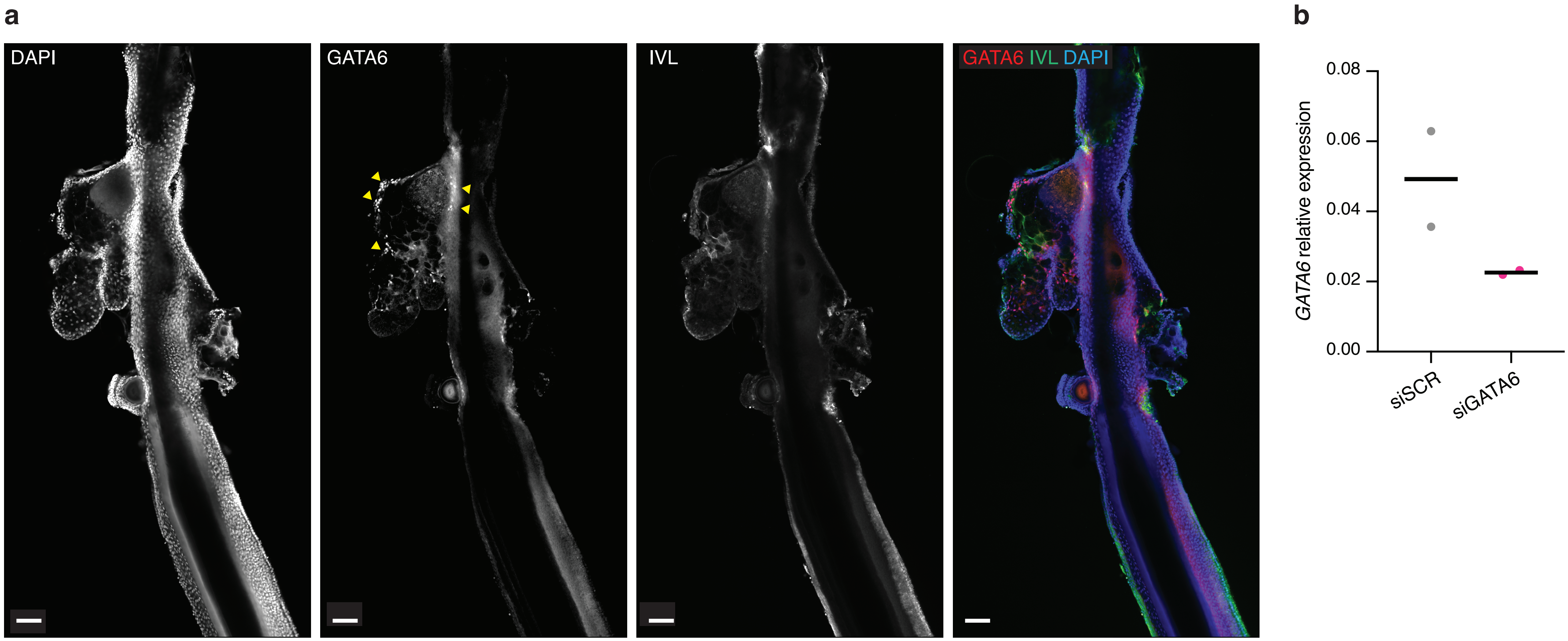

Supplementary Fig. 5: GATA6 knockdown in organ-cultured human hair follicles.
(**a**) GATA6 is expressed in the upper pilosebaceous unit in a hair follicle micro-dissected from human facial skin (yellow arrowheads). IVL expression is detected in the infundibulum. Data are representative of two independent experiments. Scale bar: 100 µm. (**b**) GATA6 expression was measured by RT-qPCR in organ-cultured human hair follicles upon siSCR or siGATA6 transfection. Data are presented as mean with individual values and were obtained from two independent experiments corresponding to n=2 replicates per condition. Source data are provided as a Source Data file.

Supplementary Fig. 6

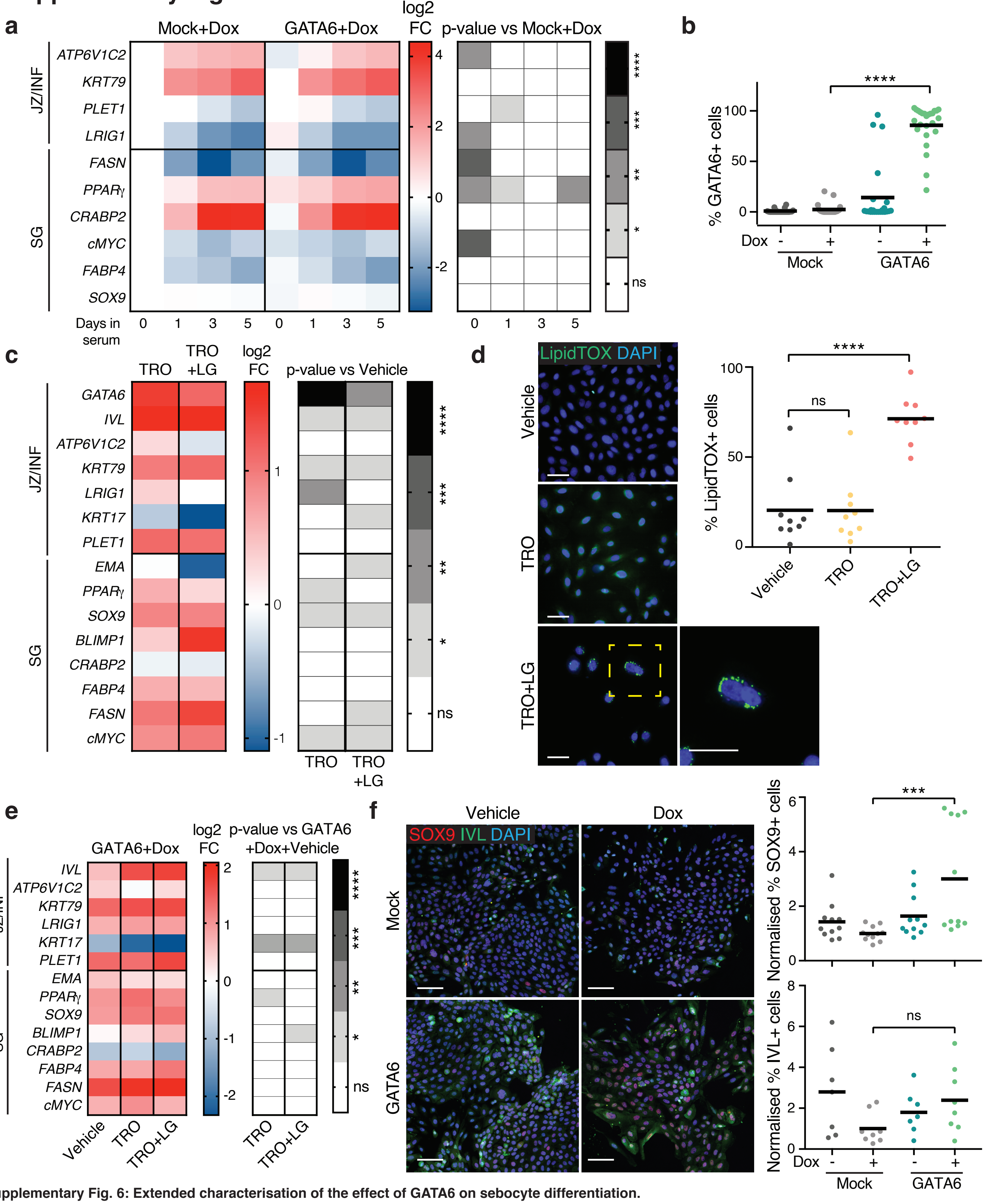

Supplementary Fig. 6: Extended characterisation of the effect of GATA6 on sebocyte differentiation. (**a**) Mock or GATA6-IFE keratinocytes were treated with 1 µg/ml Dox for 16 h and then induced to differentiate in medium containing serum and Dox for 0 (n=8/condition), 1 (n=6/condition), 3 (n=6/condition) or 5 days (n=8/condition). Expression of JZ/INF and SG markers was assessed by RT-qPCR and normalised to housekeeping gene expression. Values are expressed as log2 FC over Mock values at 0 h. (**b**) Mock or GATA6-infected sebocytes were treated with 1 µg/ml Dox or vehicle for 5 days (n=26 for Vehicle conditions, n=25 for Dox conditions; p<0.000001). % of GATA6+ cells is shown. (**c**) SebE6E7 sebocytes were treated with vehicle (DMSO), 1µM TRO or 1 µM TRO + 0.1 µM LG for 4 days. Data are presented as log2 FC versus vehicle-treated cells (n=8/condition). (**d**) Cells treated as in (c) were stained with LipidTOX (n=9/condition). % of LipidTOX+ cells is shown. Lower right panel is a higher magnification view of the yellow dashed region. (**e**) Mock or GATA6- infected SebE6E7 sebocytes were treated with 1 µg/ml Dox and with DMSO, TRO or TRO+LG as in (c). Gene expression in GATA6+Dox sebocytes is represented as log2 FC versus Mock+Dox+Vehicle sebocytes (n=3/condition). (**f**) Mock or GATA6-infected sebocytes were treated as in (b). Quantitation and representative images of SOX9 (n=11/condition; p=0.000189) and IVL (n=7 for Vehicle conditions, n=8 for Dox conditions; p=0.073208) staining are shown. (**d, f**) Nuclei were counterstained with DAPI. Scale bars: 50 µm (d), 100 µm (f). (**a-f**) Data are represented as mean (a, c, e) or mean with individual values (b, d, e) and were obtained from two (a, e) or three (b-d, f) independent experiments corresponding to n replicates. Statistical analyses were performed with two-tailed multiple t-tests (a, c) or ordinary one-way ANOVA (b, d-f). (ns) not significant; (*) p-value < 0.05; (**) p-value < 0.005; (***) p-value < 0.0005; (****) p-value < 0.00005. Source data are provided as a Source Data file.

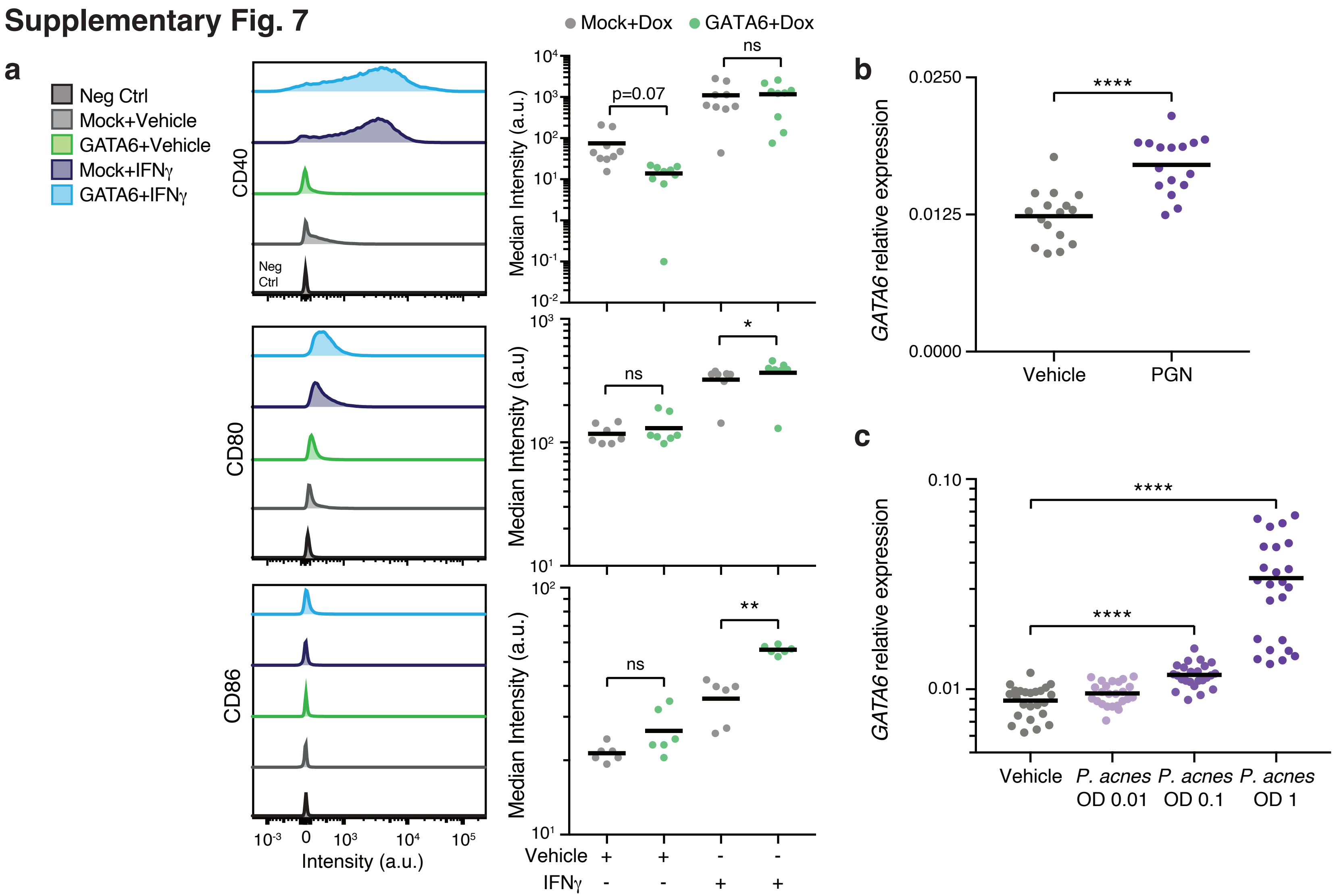

Supplementary Fig. 7: GATA6 induces a change in immunological ligand expression and is induced by bacterial insults in sebocytes.

(**a**) SebE6E7 sebocytes infected with mock or GATA6 lentiviruses were treated with 1 µg.ml^{-1} Dox and 1 U.ml^{-1} human recombinant IFNγ or vehicle for 3 days. Median intensity of CD40 (n=9/condition), CD80 (n=7/condition) and CD86 (n=6/condition) staining was analysed by flow cytometry. Representative FACS plots are shown. (**b**) Sebocytes were treated with 1 µg.ml^{-1} PGN or vehicle for 3 days (n=16/condition; p=0.000006). Expression of GATA6 was assessed by RT-qPCR and normalised to housekeeping gene expression. (**c**) SebE6E7 sebocytes were treated with cultures of *P. acnes* (OD 0.01, 0.1 or 1) for 16 h (n=24/condition). Expression of GATA6 was assessed as in (b). (**a-c**) Data are represented as mean with individual values and were obtained from four independent experiments corresponding to n replicates. Statistical analyses were performed with ordinary one-way ANOVA (a, c) or two-tailed paired t-test (b). (ns) not significant; (*) p-value < 0.05; (**) p-value < 0.005; (****) p-value < 0.00005. Source data are provided as a Source Data file.

Supplementary Fig. 8

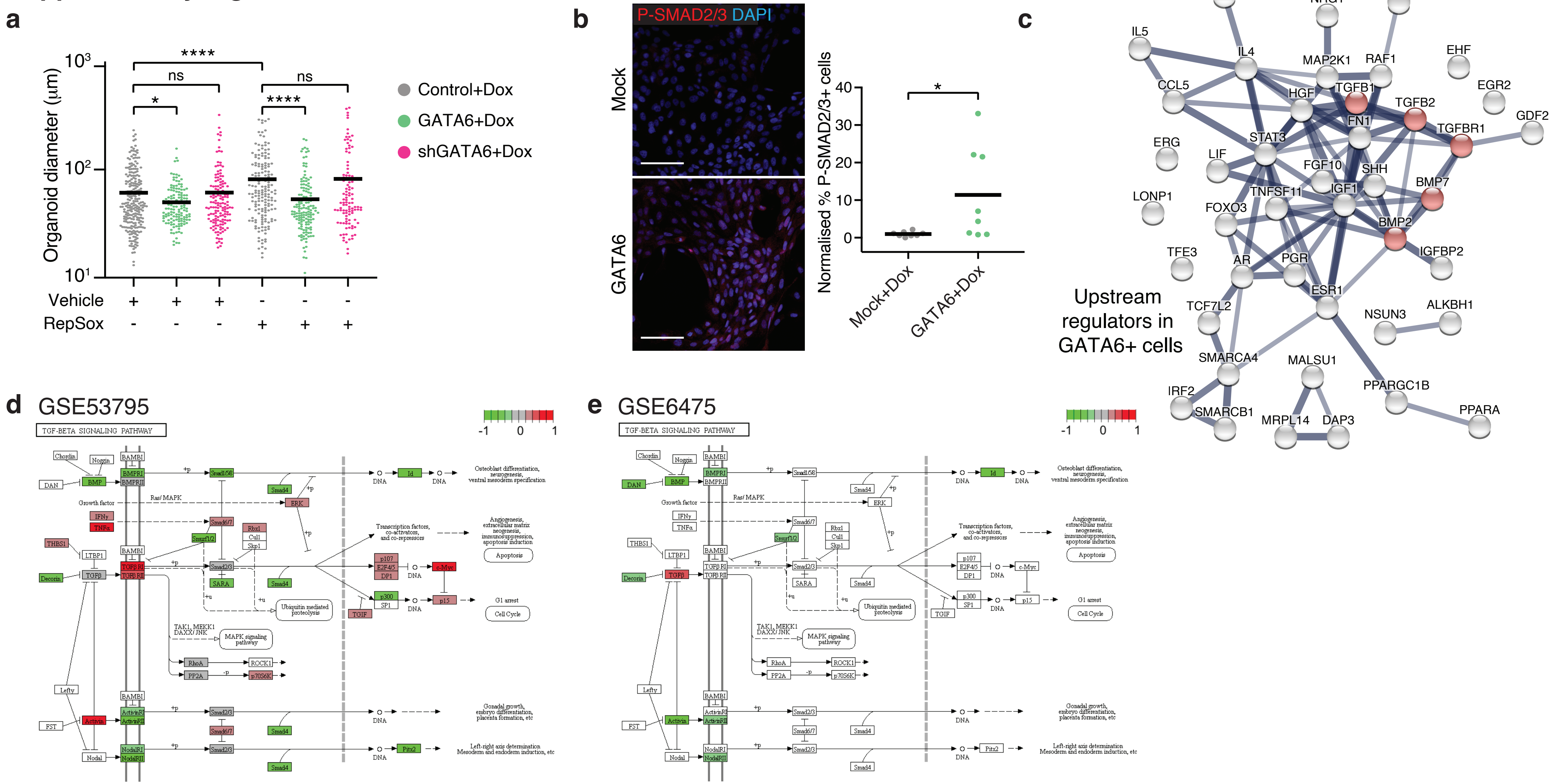

Supplementary Fig. 8: TGFβ pathway is modulated in acne disease.
(**a**) Quantification of organoid diameter of control (Mock and shSCR), GATA6, or shGATA6-expressing sebaceous organoids treated with vehicle or 25 µM RepSox as in Fig. 7b (n=37/condition). (**b**) Mock and GATA6 sebocytes were treated with 1 µg.ml^{-1} Dox or vehicle for 5 days (n=8/condition; p=0.0328). Quantitation and representative images for P-SMAD2/3 staining are shown. (**c**) The STRING functional network predicted protein associations between upstream regulators of GATA6+ DEGs. Upstream regulators were obtained from IPA Upstream Regulator Analysis of the 250 most upregulated GATA6+ DEGs. Nodes of proteins involved in KEGG pathway "TGFβ Signalling" are coloured in red. (**d-e**) Overlay of gene expression changes in TGFβ signalling pathway from acne lesions and unaffected skin in microarray datasets GSE53795 (d) and GSE6475 (e) respectively. Transcripts significantly changed ($p<0.05$) are colour-coded (green: negative FC; red: positive FC) and reported on TGFβ pathway from KEGG graph as rendered by Pathview. (**a-b**) Data are mean with individual values and were obtained from two (b) or three (a) independent experiments corresponding to n replicates. Statistical analyses were performed with ordinary one-way ANOVA (a) or two-tailed unpaired t-test (b). (ns) not significant; (*) p-value < 0.05; (****) p-value < 0.00005. Source data are provided as a Source Data file.

Supplementary Fig. 9

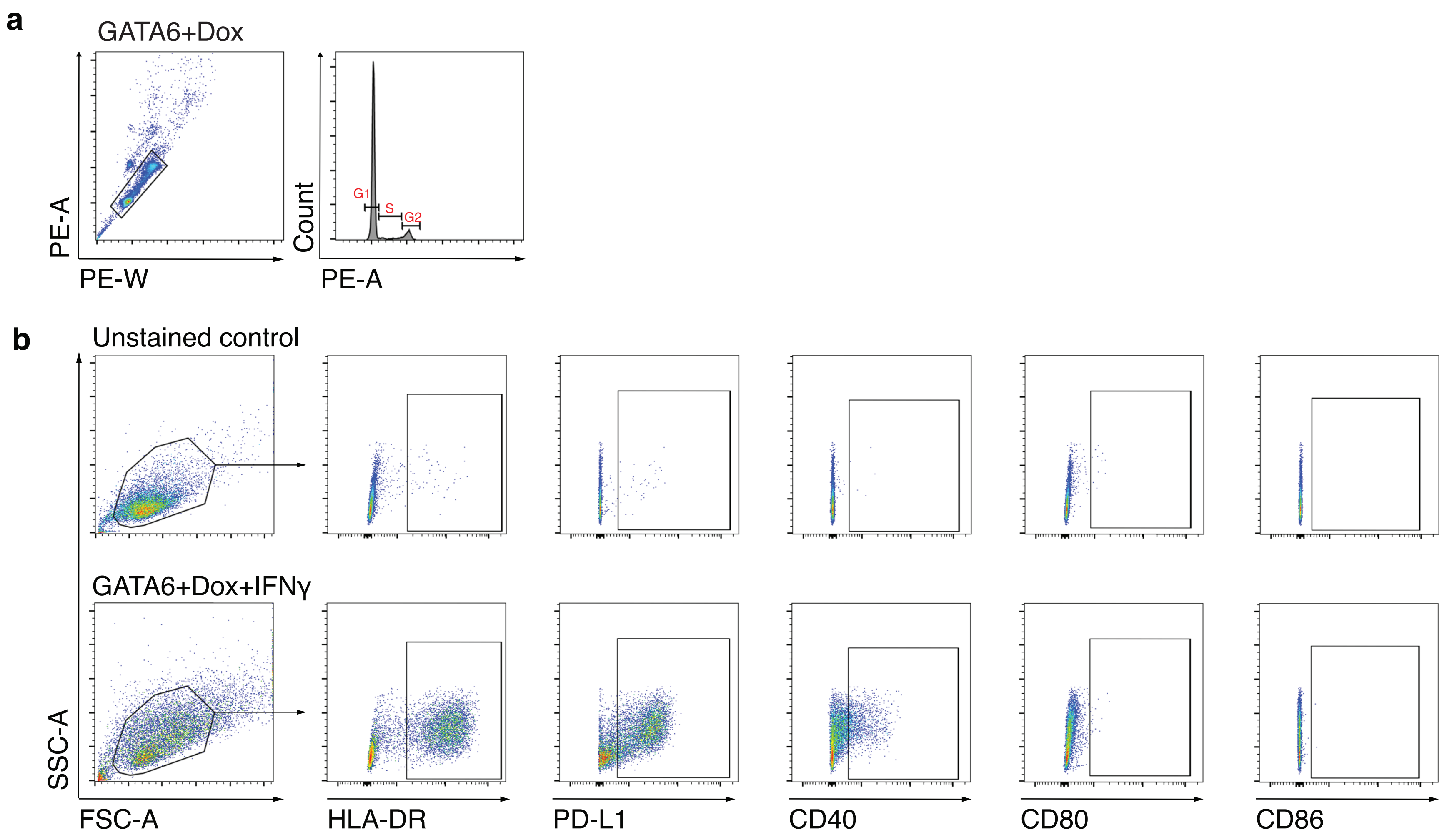

Supplementary Fig. 9: Gating strategies for flow cytometry analyses.
(**a**) Cell-cycle analysis of Mock or GATA6 infected sebocytes treated with 1 µg.ml^{-1} Dox for 4 days. Representative histograms of propidium iodide (PI)-stained cells, with singlets discrimination and PI intensity, as presented in Fig. 3e. (**b**) GATA6-overexpressing sebocytes treated with 1 µg.ml^{-1} Dox and 1 U.ml^{-1} human recombinant IFNγ or vehicle for 3 days. Expression of immune cell surface markers were measured compared to unstained control cells in Fig 6c and Supplementary Fig.7a.
