# Supplementary material for: Contribution of GATA6 to homeostasis of the human upper pilosebaceous unit and acne pathogenesis
Source: Nat Commun. 2020 Oct 20;11:5067. doi: 10.1038/s41467-020-18784-z (PMC7575575; doi:10.1038/s41467-020-18784-z)
Supplement: Supplementary file 3 — Description of Additional Supplementary Files [file 41467_2020_18784_MOESM3_ESM.docx]

**Description of Additional Supplementary Files**

**File Name: Supplementary Data 1**

Description: Differentially Expressed Genes in Cluster 22 (p-values were calculated between cluster 22 and all other cells using a two-sided Wilcoxon test).

**File Name: Supplementary Data 2**Description: Differentially Expressed Genes in *GATA6*+ cells (p-values were calculated between *GATA6*+ and *GATA6*- cells using a two-sided Wilcoxon test).

**File Name: Supplementary Data 3**Description: List of primers.

**File Name: Source Data**Description: Quantitative data used to create the figures.
