# Supplementary material for: Contribution of GATA6 to homeostasis of the human upper pilosebaceous unit and acne pathogenesis
Source: Nat Commun. 2020 Oct 20;11:5067. doi: 10.1038/s41467-020-18784-z (PMC7575575; doi:10.1038/s41467-020-18784-z)
Supplement: Supplementary file 7 — Reporting Summary [file 41467_2020_18784_MOESM7_ESM.pdf]

nature research

Corresponding author(s): Fiona M. Watt

Last updated by author(s): Aug 5, 2020

Reporting Summary

Nature Research wishes to improve the reproducibility of the work that we publish. This form provides structure for consistency and transparency in reporting. For further information on Nature Research policies, see our Editorial Policies and the Editorial Policy Checklist.

Statistics

For all statistical analyses, confirm that the following items are present in the figure legend, table legend, main text, or Methods section.

n/a	Confirmed	
☐	☒	The exact sample size (n) for each experimental group/condition, given as a discrete number and unit of measurement
☐	☒	A statement on whether measurements were taken from distinct samples or whether the same sample was measured repeatedly
☐	☒	The statistical test(s) used AND whether they are one- or two-sided<br>*Only common tests should be described solely by name; describe more complex techniques in the Methods section.*
☐	☒	A description of all covariates tested
☐	☒	A description of any assumptions or corrections, such as tests of normality and adjustment for multiple comparisons
☐	☒	A full description of the statistical parameters including central tendency (e.g. means) or other basic estimates (e.g. regression coefficient) AND variation (e.g. standard deviation) or associated estimates of uncertainty (e.g. confidence intervals)
☐	☒	For null hypothesis testing, the test statistic (e.g. F, t, r) with confidence intervals, effect sizes, degrees of freedom and P value noted<br>*Give P values as exact values whenever suitable.*
☒	☐	For Bayesian analysis, information on the choice of priors and Markov chain Monte Carlo settings
☒	☐	For hierarchical and complex designs, identification of the appropriate level for tests and full reporting of outcomes
☒	☐	Estimates of effect sizes (e.g. Cohen's d, Pearson's r), indicating how they were calculated

Our web collection on statistics for biologists contains articles on many of the points above.

Software and code

Policy information about availability of computer code

Data collection	No software was used for data collection.

Data analysis	Softwares used for data analyses were:<br>- Prism 7 and 8 software (GraphPad)<br>- Harmony high content analysis version 4.1 software package (Perkin-Elmer)<br>- ImageJ version 2.0.0-rc-69/1.52p (https://imagej.nih.gov/ij/)<br>- QuPath version 0.1.2 (https://qupath.github.io)<br>- NIS elements Viewer version 4.11.0 (Nikon)<br>- NIS elements Advanced Research version 5.11.01 (Nikon)<br>- Incucyte zoom software version 2018A (Essen)z<br>- ImageLab software version 4.1 (Bio-Rad Laboratories)<br>- Seurat R package version 2.3.0 using R version 3.6.1 (https://www.r-project.org)<br>- Ingenuity Pathway Analysis (IPA) version 01-13 (Qiagen)<br>- DAVID software version 6.8 (https://david.ncifcrf.gov)<br>- NCBI GEO2R tool (https://www.ncbi.nlm.nih.gov/geo/geo2r/)<br>- String versions 10.5 and 11.0 (https://string-db.org)<br>- Primer3Plus version 2 (http://www.bioinformatics.nl/cgi-bin/primer3plus/primer3plus.cgi)<br>- FlowJo software version 10.4 (FlowJo)<br><br>The home-made Python script developed to quantify clonogenicity assays is available in GitHub (https://github.com/MATBEO/clonogenicity_assay_script).

For manuscripts utilizing custom algorithms or software that are central to the research but not yet described in published literature, software must be made available to editors and reviewers. We strongly encourage code deposition in a community repository (e.g. GitHub). See the Nature Research guidelines for submitting code & software for further information.

Data

Policy information about availability of data

All manuscripts must include a data availability statement. This statement should provide the following information, where applicable:

- Accession codes, unique identifiers, or web links for publicly available datasets
- A list of figures that have associated raw data
- A description of any restrictions on data availability

All data that support the findings of this study are available within the paper and its supplementary information files or are available from the corresponding author upon reasonable request. Source data for Fig. 2b, Fig. 3b-g, Fig. 4a, Fig. 4c-f, Fig. 5a-f, Fig. 6a-c, Fig. 7b-e, Supplementary Fig. 3a-b, Supplementary Fig. 4a-b, Supplementary Fig. 5b, Supplementary Fig. 6a-f, Supplementary Fig. 7a-c, Supplementary Fig. 8a-b are provided with this paper as a Source Data file. Publicly available datasets used in this study were: EGAS0000100292729 (https://ega-archive.org/datasets/EGAD00010001620), GEO GSE5379531 (https://www.ncbi.nlm.nih.gov/geo/query/acc.cgi?acc=GSE53795) and GSE647530 (https://www.ncbi.nlm.nih.gov/geo/query/acc.cgi?acc=GSE6475).

Field-specific reporting

Please select the one below that is the best fit for your research. If you are not sure, read the appropriate sections before making your selection.

☒ Life sciences ☐ Behavioural & social sciences ☐ Ecological, evolutionary & environmental sciences

For a reference copy of the document with all sections, see nature.com/documents/nr-reporting-summary-flat.pdf

Life sciences study design

All studies must disclose on these points even when the disclosure is negative.

Sample size	No statistical method was used to predetermine sample size. The sample size was determined from preliminary experiments and was based on the reproducibility between independent experiments. The experiments were designed to use the smallest number of samples needed to obtain the requested data. For human samples, sample size was determined by the number of samples we were able to collect since it is infrequent to have acne biopsies in pathology department.
Data exclusions	No data were excluded.
Replication	All data were successfully replicated at least two times (excepted for Supplementary Fig. 3c that was performed only once). The number of independent experiments and replicates is indicated in the figure legends, and they were all included to perform statistical analyses.
Randomization	As our data do not include cinical trials or related experiments, randomization was not applied.<br>For experiments involving cells, all conditions were plated at the same time and all the experimental procedures were strictly similar between the different conditions.
Blinding	No blinding method was possible as only one experimenter was performing the analyses.

Reporting for specific materials, systems and methods

We require information from authors about some types of materials, experimental systems and methods used in many studies. Here, indicate whether each material, system or method listed is relevant to your study. If you are not sure if a list item applies to your research, read the appropriate section before selecting a response.

Materials & experimental systems

n/a	Involved in the study
☐	☒ Antibodies
☐	☒ Eukaryotic cell lines
☒	☐ Palaeontology and archaeology
☒	☐ Animals and other organisms
☐	☒ Human research participants
☒	☐ Clinical data
☒	☐ Dual use research of concern

Methods

n/a	Involved in the study
☒	☐ ChIP-seq
☐	☒ Flow cytometry
☒	☐ MRI-based neuroimaging

Antibodies

Antibodies used	Primary antibodies were used at the indicated dilutions for immunofluorescence studies: GATA6 (1:200, D61E4 clone, Cell Signalling 5851); ITGa6 (1:200, GoH3 clone, eBioscience 14-0495-82); IVL (1:200, clone SY7 clone, CRUK); FASN (1:100, Santa Cruz sc-48357); KRT7 (1:100, LK1K clone, Thermo Fisher Scientific MA1-90894); KRT14 (1:200, Covance, SIG-3476-100); PanKer (1:400, clone LP34, LSBio LS-C95318); LOR (1:200, Covance, PRB145P); PLET1 (1:100, 1D4 clone, EMD Millipore MAB4416); P-SMAD2/3 (Phospho-SMAD2 (Ser465/467) / SMAD3 (Ser423/425)) (1:100, D27F4 clone, Cell Signaling 8828); P-SMAD2 (Phospho-SMAD2 (Ser465/Ser467)) (1:100, E8F3R clone, Cell Signaling 18338); KRT79 (1:100, Santa-Cruz, sc-243156); PPARg (1:100, E-8 clone, Santa Cruz, sc-7273); LRIG1 (1:50, R&D Systems, MAB7498); SOX9 (1:100, R&D Systems, AF3075); BLIMP1 (1:100, 6DE clone, eBioscience 14-5963-82); AR (1:100, AN1-15 clone, Santa Cruz, sc-56824); KI67 (1:100, 8D5 clone, Cell Signaling 9449).<br>Primary antibodies were used at the indicated dilutions for histopathological analyses: GATA6 (1:750, D61E4 clone, Cell Signalling 5851), Ki67 (1:100, Clone MIB-1, Dako M7240) and KRT5/6 (1:100, Clone D5/16 B4, Dako M7237).<br>For FACS, the following primary antibodies were used: CD86 - PE (1:50, IT2.2 clone, Biolegend, 305405); CD40 - APC/ Cy7 (1:50, 5C3 clone, Biolegend, 334323); HLA-DR - Pacific Blue (1:50, LN3 clone, Biolegend, 327016); CD80 - Brilliant Violet 605 (1:50, 2D10 clone, Biolegend, 305225); CD274/PD-L1/B7-H1 - PE-Cy7 (1:50, MIH1 clone, eBioscience, 25-5983-41).
Validation	All primary antibodies used in this study were validated by manufacturer companies. Validation data / citations can be found on manufacturers websites by searching the antibody catalog number provided in materials and methods section of our manuscript.

Eukaryotic cell lines

Policy information about cell lines

Cell line source(s)	Primary human neonatal IFE keratinocytes (strain km) and foetal outer root sheath keratinocytes (strain k12026) were isolated in the laboratory. The SebE6E7 sebocyte cell line was generated in the laboratory from sebaceous glands micro-dissected from adult human facial skin and immortalized by retroviral transduction of HPV16/E6E7 genes (32). 3T3-J2 cells were a gift from James Rheinwald and can be purchased from Kerafast (EF3003). HEK-293 cells were purchased from ATCC (CRL-1573).
Authentication	No authentication method was used.
Mycoplasma contamination	All cell stocks were routinely tested for mycoplasma contamination and were negative.
Commonly misidentified lines (See ICLAC register)	No commonly misidentified cell lines were used in the study.

Human research participants

Policy information about studies involving human research participants

Population characteristics	Our research involving human participants only includes histopathological analyses of healthy and acne skin. The diagnosis of healthy or acne skin was made based on the H&E coloration. We did not collect covariate population characteristics (such as age, gender, genotypic information, past and current diagnosis and treatment categories) as we did not perform interventional studies and as this would not have modified our analyses.
Recruitment	Samples of adult healthy skin (surplus surgical waste) were obtained from patients undergoing plastic surgery whenever they agreed to donate a sample of skin for research after informed consent.<br>Acne vulgaris samples were obtained from diagnostic biopsies retrieved from pathology departments archives of Hôpitaux Universitaires Paris Nord Val de Seine. To identify these samples, a search was performed in the department archives for biopsies diagnosed as acne.
Ethics oversight	All human tissue samples were collected after informed consent and processed in compliance with all relevant ethical regulation, including the Declaration of Helsinki's recommendations on human research and the UK Human Tissue Act (2004).<br>Samples of adult healthy skin (surplus surgical waste) were obtained from patients undergoing plastic surgery. Collection for research use of adult healthy skin was approved by the National Research Ethics Service (UK) (Human Tissue Authority Licence No. 12121, Research Ethics Committee No. 14/NS/1073).<br>Neonatal primary keratinocytes were isolated from surgically discarded foreskin from patients undergoing circumcision. This procedure was approved by the National Research Ethics Service (UK) (Research Ethics Committee No. 08/H0306/30).<br>To isolate foetal primary outer root sheath (ORS) keratinocytes, foetal back skin was obtained with appropriate ethical approval from the UK Human Developmental Biology Resource (www.hdbr.org).<br>Acne vulgaris samples were obtained from diagnostic biopsies retrieved from pathology departments archives of Hôpitaux Universitaires Paris Nord Val de Seine . For all patients, the study was performed after establishing the absence of registered opposition to the use of tissue samples according to the guidelines of the French Bioethics Law for retrospective noninterventional research studies. For all cases, there was enough formalin-fixed paraffin embedded material remaining after the diagnosis had been established to qualify the samples for additional immunohistochemical studies.

Note that full information on the approval of the study protocol must also be provided in the manuscript.

Flow Cytometry

Plots

Confirm that:

- [x] The axis labels state the marker and fluorochrome used (e.g. CD4-FITC).
- [x] The axis scales are clearly visible. Include numbers along axes only for bottom left plot of group (a 'group' is an analysis of identical markers).
- [x] All plots are contour plots with outliers or pseudocolor plots.
- [x] A numerical value for number of cells or percentage (with statistics) is provided.

Methodology

Sample preparation	For cell cycle analysis, isolated sebocytes were fixed with ice-cold 70% EtOH for 30 min at 4°C, washed twice with PBS and treated with 50 ul of 100 ug/ml RNAse A solution (Sigma-Aldrich) for 15 min at room temperature. Cells were then passed through a 70 um strainer and incubated with 200 ul of 50 ug/ml propidium iodide solution (Sigma-Aldrich) for 10 min at room temperature.<br>For immune receptors/ligands analysis, disaggregated cells were resuspended in FACS buffer (3% foetal bovine serum and 1 mM EDTA in PBS) and blocked with 5ul Human TruStain FcX (Fc Receptor Blocking Solution) (BioLegend) for 20 min at 4°C. The following primary antibodies were incubated with the cells for 20 min at 4°C in FACS buffer: CD86 - PE (1:50, IT2.2 clone, Biolegend, 305405); CD40 - APC/ Cy7 (1:50, 5C3 clone, Biolegend, 334323); HLA-DR - Pacific Blue (1:50, LN3 clone, Biolegend, 327016); CD80 - Brilliant Violet 605 (1:50, 2D10 clone, Biolegend, 305225); CD274/PD-L1/B7-H1 - PE-Cy7 (1:50, MIH1 clone, eBioscience, 25-5983-41). After incubation, cells were washed twice in FACS buffer and passed through a 70 um strainer.
Instrument	Measurements were performed with a BD FACSCanto II cell analyser and a LSRFortessa cell analyzer.
Software	Data analysis was performed using FlowJo software version 10.4 (FlowJo).
Cell population abundance	We did not perform sorting.
Gating strategy	For gate setting and compensation, unlabelled cells and single-labelled BD CompBeads (BD Biosciences) were used as controls. FMO controls were also used for gating accuracy.

- [x] Tick this box to confirm that a figure exemplifying the gating strategy is provided in the Supplementary Information.
